# Chronic heart failure following hemorrhagic myocardial infarction: mechanism, treatment and outlook

**DOI:** 10.15698/cst2023.02.276

**Published:** 2023-02-13

**Authors:** Shing Fai Chan, Keyur Vora, Rohan Dharmakumar

**Affiliations:** 1 Krannert Cardiovascular Research Center, Indiana University School of Medicine/IU Health Cardiovascular Institute.

**Keywords:** myocardial infarction, intramyocardial hemorrhage, heart failure, deferiprone, fatty infiltration, iron

## Abstract

Myocardial infarction (MI), the blockage of arterial blood supply of the heart, is among the most common causes of death worldwide. Even when patients receive immediate treatment by re-opening blocked arteries, they often develop chronic heart failure (CHF) in the aftermath of MI events. Yet, the factors that contribute to the development of MI-associated CHF are poorly understood. In our recent study (Nat Commun 13:6394), we link intramyocardial hemorrhage, an injury which can occur during reperfusion of areas affected by MI, to an increased risk of CHF. Mechanistically, our data suggest that an iron-induced adverse cascade of events after hemorrhagic MI drives fatty degeneration of infarcted tissue, which ultimately contributes to negative cardiac remodeling. In this Microreview, we discuss the implications of our findings regarding the molecular mechanism, more targeted treatment options as well as perspectives in the clinical care of CHF after hemorrhagic MI.

## CHRONIC HEART FAILURE (CHF) AND HEMORRHAGIC MYOCARDIAL INFARCTION ARE INTIMATELY RELATED

Acute myocardial infarction (AMI), from sudden obstruction of a coronary artery, afflicts approximately 1M people in the US yearly. Prompt restoration of blood flow through the epicardial arteries (reperfusion) has been a major advance, which has reduced immediate death from AMI. However, in the post-AMI period, adverse LV remodeling can result in chronic heart failure (CHF), or malignant ventricular arrhythmias can precipitate sudden cardiac arrest, both of which can increase morbidity and mortality.

The incidence of post-MI CHF has increased in recent decades. In the US, ∼2.1M patients are affected, and >250,000 new cases are reported every year. According to the Centers for Disease Control, >300,000 deaths/yr. are due to CHF. The terminal recourse of these patients is heart transplantation, which is limited by donor hearts, eligibility, and cost.

**Figure 1:**
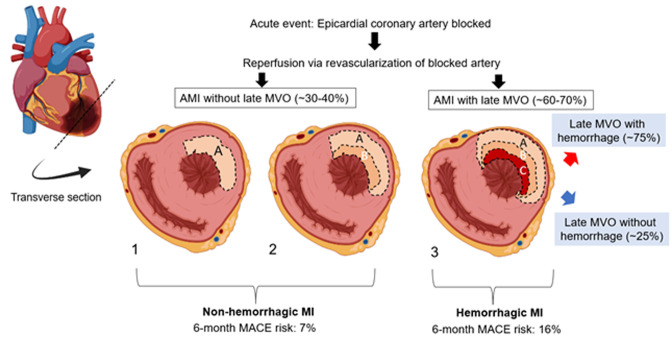
FIGURE 1: Schematic differentiating reperfused acute MIs, fraction of MIs with hemorrhage, and associated MACE risk. Myocyte death proceeds from the subendocardium as a wave of injury with increasing ischemic time with as: 1: Early, reperfusion with myocyte injury only; 2: myocyte injury with mild microvascular obstruction (MVO); 3: myocyte injury with late/persistent MVO. Zones A: myocyte injury only; B: myocyte injury and mild MVO (some slow flow); C: myocyte injury with late MVO (no flow). Intramyocardial hemorrhage occurs in ∼75% of the time in Zone C. hMIs have the largest 6-month MACE risk (16% vs. 7%), among all MI types. Figure was generated using Biorender.

Not all myocardial infarctions (MI) are the same. **Fig. 1** shows a schematic of various MI types based on the “wave front hypothesis”. Notably, >60% of AMIs have late (persistent) microvascular obstruction (MVO) resulting in “no-reflow” (zone C), despite re-establishing flow in the culprit artery. MI size is an established independent predictor of major adverse cardiac events (MACE: death, hospitalization for CHF and adverse LV remodeling). With advances in imaging, particularly cardiac magnetic resonance imaging (CMR), late MVO has also emerged as a key risk factor for MACE. Hemorrhage is present in ∼75% of MIs with late MVO, conferring >50% increased risk for MACE over MIs with late MVO but no hemorrhage. Thus, hemorrhagic MIs (hMIs) present the greatest risk for MACE among all MI patients.

Currently available post-MI medications are not specific to patients with late MVO or hemorrhage and have not been beneficial to these patients over other MI types. Several mechanical/post-conditioning treatment options to reduce late MVO during reperfusion have been investigated but their benefit has not been consistent. Several pharmacological interventions at the time of reperfusion have also been investigated but none has been able to significantly reduce MACE. Moreover, late MVO limits the influx of inflammatory cells into the MI and thereby delays infarct healing in the sub-acute phase. But how this contributes to continuous infarct remodeling throughout the chronic period, especially after late MVO resolves, is unknown. Recent studies have shown that (i) hMIs lead to persistent iron deposition within MI, (ii) new macrophages are recruited to the site of iron, and (iii) iron within MI is an independent risk factor for adverse remodeling in the chronic period in animals and patients – providing a strong correlation among hMI, iron deposition, inflammation, and adverse remodeling. These findings support the notion that hemorrhage may be a key driver of myocardial damage in reperfused MIs; however, whether hemorrhage is causally linked to adverse remodeling was unknown until the recent publication which is incorporated herein by reference.

## MECHANISM: HEMORRHAGIC INFARCTION DRIVES FATTY INFILTRATION AND CHRONIC HEART FAILURE

We hypothesized that the iron from hemorrhagic MI drives fatty transformation of infarcted tissue from continuous iron-induced macrophage activation, lipid oxidation, foam cell formation, ceroid production, foam cell apoptosis and iron recycling, which progressively drives loss of cardiac function.

Iron metabolism is tightly controlled and balanced by complex signaling networks. Macrophage, a key immune cell, plays critical role in iron homeostasis, and its regulatory mechanisms are increasingly recognized in cardiovascular disease. Dysregulation of intracellular iron metabolism in macrophages can lead to marked phenotypic and functional changes. These changes include the promotion of pro-inflammatory state, acceleration of lipid accumulation and differentiation into foam cell formation.

**Figure 2:**
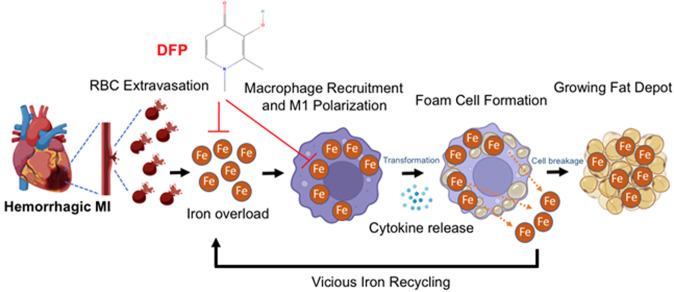
FIGURE 2: A proposed scheme of how hemorrhagic myocardial infarction (MI) induces iron accumulation, proinflammatory response and lipomatous metaplasia. In hemorrhagic myocardial infarction (MI), the extravasated red blood cells (RBCs) lead to iron overload and promote the recruitment of unpolarized macrophages to the infarction zone. This iron when taken up by macrophages induce their polarization into pro-inflammatory state and subsequently transformation into foam cells through disproportionate lipid influx and cholesterol accumulation. As the disease progresses, iron is released through foam cell degradation (apoptosis), which enters a vicious cycle that perpetuates to continually support inflammation and leads to the growth of the fat depot in the infarction zone. These detrimental events contribute to functional and structural losses of the heart, which define chronic heart failure. Timely application of an intra- and extra-cellular ferric iron chelator deferiprone (DFP) can markedly reduce iron and fat with the MI zone. Figure was generated using Biorender.

Our recent work shows that excessive iron from intramyocardial hemorrhage is a deleterious contributor to the development and progression of adverse outcomes following hMI. Excessive iron within the MI zone results in the oxidation of the lipids in the infarct zone and promotes the recruitment of unpolarized macrophages into the vicinity. The oxidized lipids and iron are taken up by the macrophages, which increase their polarization into a pro-inflammatory state through enhanced stimulation of cytokine release. The iron within macrophages become cytotoxic when the extent of iron reaches pathological level, polarizing the macrophages to a proinflammatory state. During the development and progression of this process, the pro-inflammatory macrophages are transformed into foam cells when the intracellular lipid content of macrophages exceeds their capacity to maintain lipid homeostasis. Excessive lipid influx via scavenger receptors occurs within the areas of hMI. These foam cells exhibit impaired immune function and contribute to the pathogenesis of lipomatous metaplasia by continually inducing inflammation, tissue damage and remodeling in the heart. These foam cells produce ceroids, destabilize lysosomes and eventually drive foam cell apoptosis. The remnants from foam-cell apoptosis (particularly the lipids and iron) are recycled back to enter a pathway which perpetuates inflammation, formation of foam cells and enlargement of the fat depot (**Fig. 2**).

Recent research has shown that foam cells are associated with chronic inflammation in cancer, metabolic, infectious, and autoimmune diseases. Our work suggests that the uncontrolled release of cytokine and the expansion of fat depot from apoptotic foam cells can drive inflammation and cardiac remodeling that drive progressive damage in the heart. Thus, foam cells may offer a novel putative target for pharmacological intervention against the negative contribution from intramyocardial hemorrhage. One strategy is to mitigate both chronic inflammation and fat deposition, possibly by reducing the induction and formation of foam cells in hMI. Several anti-cytokine therapies are currently under investigation as potential targets to reduce inflammation and improve cardiac function in acute MI or chronic HF. However, none of these therapies have been investigated in the setting of hMI. Previous studies have found that inhibition of interleukin-1 (IL-1), -6 (IL-6), -8 (IL-8), monocyte chemoattractant protein-1 (MCP-1), CC- and CXC- chemokines, or tumor necrosis factor-α (TNF-α) have had beneficial effects on cardiac function. However, neutral and detrimental results have also been reported for some anti-cytokine therapies of IL-1, IL-6, IL-8, and MCP-1. Human clinical studies are currently limited to IL-1 β inhibition, IL-1 receptor antagonists (IL-1RA), IL-6 receptor antagonists (IL-6RA) or TNF-α inhibition, but most prospective studies unfortunately have reported disappointing and inconsistent results. Moreover, a recent study has demonstrated that infiltration of intramyocardial fat within the scarred infraction zone can be a source of infarct-related ventricular arrhythmias (VT) in the chronic stage of MI. Previous studies have also shown that hMI patients may be at greater risk of developing malignant ventricular arrhythmias, which is a critical contributor to sudden cardiac arrest and sudden cardiac death in chronic MI patients. However, whether the initiation of VTs in hMI patients is a consequence of hemorrhage-related iron driving lipomatous metaplasia remains an open question.

## PAST POST-MI THERAPIES DID NOT TARGET HEMORRHAGIC MYOCARDIAL INFARCTION

Oxidative stress has been identified to be a key mechanism of reperfusion injury. This led to anti-oxidant therapies to curb the damaging effects of oxidative stress in AMI, but the results have been mixed. Recent Trial to Assess Chelation Therapy (TACT) in post MI patients showed that 6 months of EDTA therapy starting 6-weeks post MI did not substantially decrease MACE. It is to be noted however that EDTA is (a) not specific for ferric iron and (b) cannot cross cell membranes. Further, hemorrhage-derived iron is intracellular and trivalent, and that TACT did not stratify patients for hMI.

Prior studies evaluating iron-chelation therapy (ICT) in the post MI setting were limited to 1-2 days post MI with less than favorable outcomes. Notably, these studies did not (i) target hMI; (ii) adequately dose the subject; (iii) consider the size of the iron chelators; or (iv) confirm/quantify the clearance of iron from MI territories. ICTs are routinely used clinically to treat iron overload cardiomyopathies (thalassemia, Friedrich's ataxia, etc.). Outside of systemic iron overload, ICTs are rapidly gaining importance for their capabilities to reduce infarct expansion in ischemic strokes, neurodegenerative decline, and relief from cerebral malaria. However, outside of our study and another study in a swine model, iron chelators have not targeted hMIs to prevent heart failure. Our findings reveal that hMI zone has an elevated and stable level of iron level across a 6-month period. Notably, we found that there is a significant positive correlation between iron accumulation in hMI and fat deposition throughout the chronic phase of MI, suggesting that iron concentration within the MI zone is a primary determinant contributing to the extent of fat deposition within the infarct territory. Our work also supports the notion that an alternative therapeutic approach, one that is based on reduction of iron within the hMI zone can be beneficial.

## TREATMENT: DEFERIPRONE THERAPY POST HEMORRHAGIC MI REDUCES IRON AND FAT INFILTRATION AND RE-DIRECTS THE HEART TOWARDS FAVORABLE REMODELING

**Figure 3:**
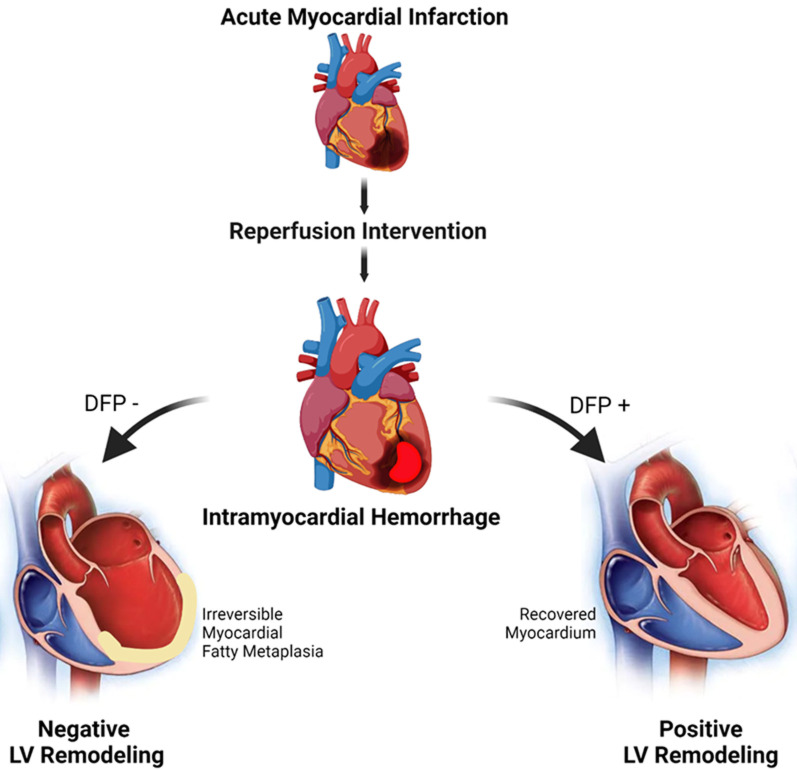
FIGURE 3: Schematic of differential LV remodelling post hemorrhagic MI with and without oral administration of deferiprone. Iron deposits from post-reperfusion intramyocardial hemorrhage drives the negative ventricular remodeling mechanistically towards fatty degeneration. However, intervention of deferiprone disrupts the iron-mediated degeneration and drives the heart towards positive remodeling. The area of MI is shown as black area on left ventricle in the top image, intramyocardial hemorrhage is shown as red area on image second from the top; negative left-ventricular remodeling is depicted by dilated left ventricle on DFP-image with semicircular white area representing lipomatous metaplasia; DFP+ image is shown with normal sized LV. Figure was generated using Biorender.

Our studies demonstrate that deferiprone (DFP), an FDA-approved iron chelator, when administered days after reperfusion, reduces iron towards baseline and lipomatous metaplasia within hemorrhagic MI territories, and mitigates adverse cardiac remodeling. Deferiprone is an intracellular and extracellular ferric iron chelator, which has a proven clinical track record for removing iron from cardiomyocytes for mitigating iron overload cardiotoxicity in thalassemia patients. Our preclinical trial showed that oral administration of DFP in the setting of reperfused hMI can also significantly reduce the hemorrhage-derived iron and halt the vicious cycle of iron-mediated cardiotoxicity. In our study, the oral administration of DFP for 8 weeks post MI showed favorable reduction in iron and fat content measured in kinematic fashion by cardiovascular magnetic resonance (T2* and confounder corrected proton-density fat fraction, respectively). In relation to the untreated group, DFP promoted positive LV remodeling as characterized by structural and functional parameters. Most notably DFP treatment improved LVEF by 36% as compared to untreated hMI subjects. Thus, an iron chelator such as DFP has the potential to limit or even regress the progression of adverse effects associated with hMI (**Fig. 2 and 3**). Importantly, our findings redefine the conventional wisdom regarding pathogenesis of hemorrhagic MI, implicating excessive iron and the associated introduction of foam cell into the chronic phase of infarction as new and important players in the pathogenesis towards post-MI heart failure. We envision that this new knowledge will be an impetus for further studies on DFP as well as the development of novel, patient-specific therapeutic strategies that target iron and fat depot to better address adverse consequences associated with hMI.

## OUTLOOK

This article highlights the clinical significance of intramyocardial hemorrhage as a significant complication of ischemia-reperfusion injury in acute myocardial infarction. Clinical investigations are now in high need to explore the translational mechanisms of intramyocardial hemorrhage mediated cardiotoxicity and potential therapeutic strategies. Driven by the recent findings, deferiprone is currently being investigated in a first-in-human trial (MIRON-DFP; NCT05604131) for the treatment of hemorrhagic MI. The current study and larger-scale clinical trials are expected to provide evidence on prevention of adverse structural LV remodeling culminating in chronic heart failure secondary to hemorrhagic MIs.

